# Root canal length estimated by cone-beam computed tomography at different slice thicknesses, dedicated endodontic software, or measured by an electronic apex locator

**DOI:** 10.1038/s41598-022-10534-z

**Published:** 2022-04-20

**Authors:** Van-Khoa Pham, Tran-Lan-Khue Pham

**Affiliations:** 1 Department of Operative Dentistry and Endodontics, Faculty of Odonto-Stomatology, University of Medicine and Pharmacy at Ho Chi Minh City, Ho Chi Minh City, Vietnam; 2 Faculty of Odonto-Stomatology, University of Medicine and Pharmacy at Ho Chi Minh City, Ho Chi Minh City, Vietnam

**Keywords:** Endodontic instruments, Apex locators

## Abstract

The aim of the present study was to evaluate the agreements between the root canal length estimations using cone-beam computed tomography (CBCT) at different slice thicknesses, dedicated software, or an electronic apex locator (EAL) and the actual lengths (AL). In total, 111 extracted human molars with 302 root canals were chosen. Teeth were scanned using a CBCT device at a voxel size of 0.075 mm. Root canal lengths were estimated using CBCT software at different slice thicknesses (0.6, 1.2, and 2.4 mm) and dedicated software for proposed or operator lengths. The endodontic access cavities were created, and root canal lengths were estimated with an EAL for electronic length (EL) and a ruler for AL. Data were tested using paired t-tests and Bland–Altman plots to detect the differences between the methods in length estimation at a significance of 0.05. The accuracy in the range of ± 0.5 mm was 100% for the EAL. There was an agreement between the EL and CBCT at a slice thickness of 1.2 mm (*p* = 0.349). CBCT at the smallest slice thickness estimation was not the best modality in agreement with the AL. The EAL was an accurate and reliable method for root canal length measurement.

## Introduction

Endodontic working length (WL), which is established on the root canal length, is one of the most important parameters in endodontic preparation^1^. An appropriate WL is of utmost importance in keeping the preparation inside the restricted radicular space, in order to avoid apical extrusion and secure good obturation^1^. Although apical constriction is the ideal and practical point where the root canal procedure should end, this anatomical landmark does not exist in every case^2^. Regardless of the existence or absence of this inconsistent feature, effort should be incessantly pursued to confine endodontic therapy to within the root canal limits. Locating the endpoint of the root canal space or apical constriction has never been easier than with the clinical modalities that are commonly used today^3^.

‘Multiple frequencies’ or the ‘ratio method’ is the dominant technique used by the contemporary electronic apex locator (EAL), after several technologies were unsuccessfully developed^4^. Although modern EAL performance might be considerably affected by certain conditions, such as the electric conductivity of metal, lack of canal patency, or complexities in anatomical structure^5^, it has outstanding advantages over periapical radiograph and in certain circumstances, the latter would seem to be dispensable^3^.

Cone-beam computed tomography (CBCT) has gradually become a more popular modality in endodontic treatment^6^; however, by following the ALARA principle, the patient would not benefit from high radiation exposure^3,7^. There are many advantages obtained from CBCT data, including exact dimensions and three-dimensional visualisation, the enhanced observation of roots and/or surrounding landmarks and the detection of peri-radicular pathology, including root resorption or root fractures^6^. The gauging tools included in the software are helpful in the determination of intra- and intercanal distances and exact measurements of root canal length^8^. Reports from previous studies on the accuracy of CBCT measurements compared with that of periapical radiographs or EALs are not in agreement^1,9,10^.

Slice thickness is an important factor in CBCT, along with voxel size, field of view and the reconstruction algorithm^11^. Slice thickness with a larger voxel size improves the contrast of images, facilitates the identification of anatomical structures and affects the accuracy of the measurement of certain distances^11,12^. Some studies have shown effects of slice thickness on the accuracy of specific issues, such as linear measurements of titanium pins, implant sites, occlusal caries, intact buccal cortical plates, or different devices^12–16^. To date, there are no data on the accuracy of root canal length measurements at different slice thicknesses.

Recently, a dedicated software package (3D Endo; Dentsply Sirona, Johnson, TN, USA) was introduced for complex case planning in endodontic treatment^17^. The software uses input CBCT data in standard DICOM with a minimum resolution of 200 µm and offers an intuitive and attractive interface for analysis. With simple instructions, the operator can semiautomatically create a canal pathway from the orifice to the apical foramen in all three spatial planes. From this step, the proposed length or correct length of the canal can be calculated by the software and displayed on the screen of the computer^18–20^.

The aim of the present study was to evaluate the agreement between root canal length estimations using CBCT (Romexis Viewer; Planmeca Oy, Helsinki, Finland) at different slice thicknesses and using dedicated software, or the EAL (RomiApex A-15; Romidan, Kiryat Ono, Israel), in comparison with actual root canal lengths.

The null hypothesis (H_0_) was that there would be agreements between root canal length estimations using different experimental methods and the actual root canal length, i.e., there would be no statistically significant differences between root canal length estimations using different methods and the actual root canal length.

## Methods

The present study was approved by the Institutional Review Board of the University of Medicine and Pharmacy at Ho Chi Minh City, Vietnam. The approval number of the study was 20,164-ĐHYD on 21st April 2020. All methods were carried out in accordance with relevant guidelines and regulations^21^. The study acquired intact extracted human molars obtained from many hospitals for numerous reasons, with informed consent obtained from all participants. With the data from a previous study^1^, the sample size was 302 root canals in 111 molars, according to calculations performed using statistical software (MedCalc Statistical Software version 19; MedCalc Software, Ostend, Belgium). The parameters for the Bland–Altman plot sample size window of the software were an alpha of 0.05, beta of 0.01, expected mean difference of 0.36, expected standard deviation of 0.31 and maximum allowed difference between methods of 1.1. The present study was performed following the method of a previous study^19^. Briefly, teeth were ultrasonically cleaned with an ultrasonic scaler (BobCat; Dentsply Sirona, Ballaigues, Switzerland). Teeth were thoroughly observed using a stereomicroscope to exclude defective teeth, such as those with an immature apex, external resorption, or a cracked root at a magnification of 10×. Teeth were coded with numbers on the coronal parts and were then placed in a plastic impression mould containing light silicone with a 3 mm wax layer on the floor. The plastic mould with teeth was then scanned using CBCT (Planmeca ProMax; Planmeca Oy, Helsinki, Finland) in endo mode, 90 kV, 10 mA, field of view 50 × 50 mm^2^ and a voxel size of 0.075 mm.

For the first part, the CBCT images were analysed using software from the CBCT manufacturer (Romexis Viewer; Planmeca Oy, Helsinki, Finland). From the slice thickness box of the software interface, different slice thicknesses were selected to measure the root canal length at three thicknesses, i.e., 0.6, 1.2 and 2.4 mm. The following steps were performed on the CBCT software.

**Figure 1:**
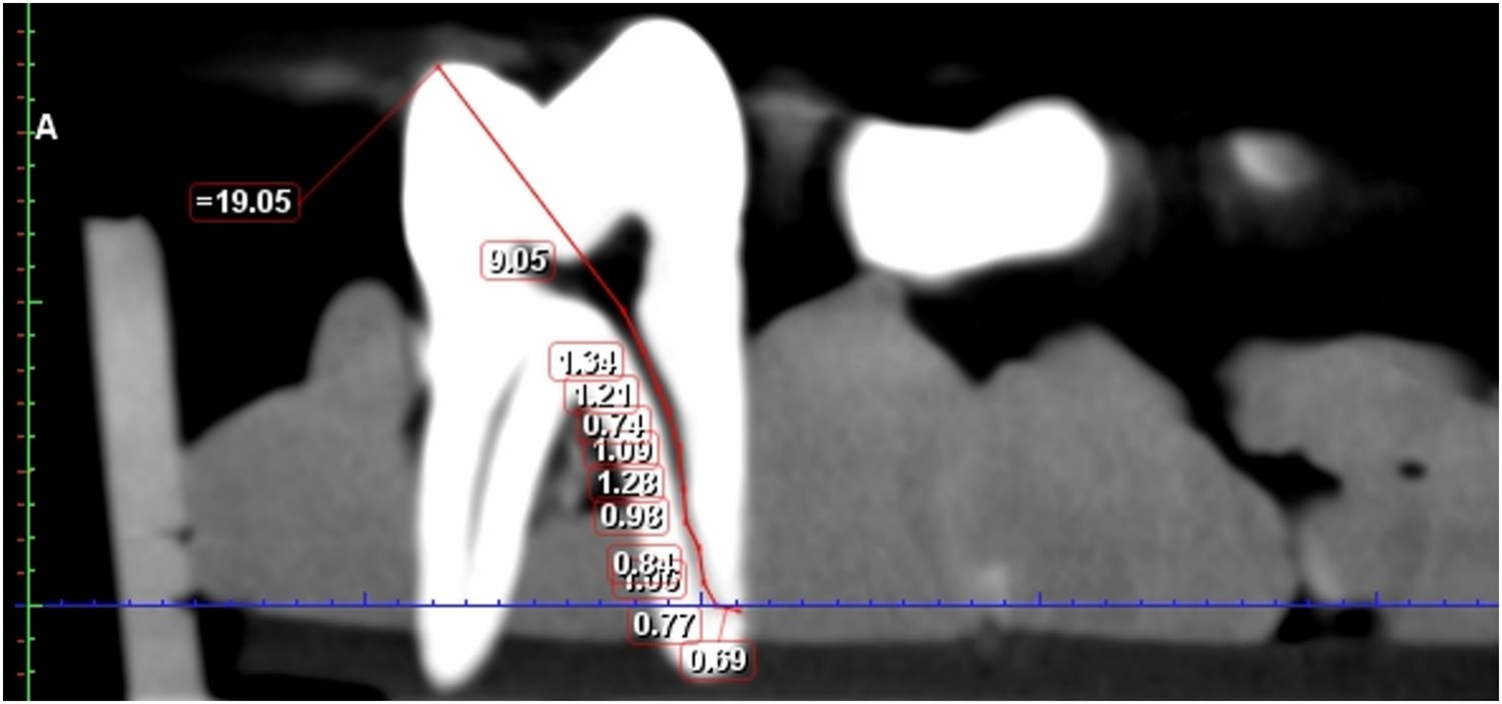
CBCT measurements of root canal length at slice thickness of 1.2 mm.

In the bucco-lingual aspect, the clearest figure of the entire canal with the largest curved angle, from the orifice to the apex, was chosen by scanning every slice of the tooth at each slice thickness in the evaluation. The apical foramen (AF) was determined by the end of the canal, which was located at the root surface on the image, and the occlusal reference (OR) was located at the farthest and most occlusal position for the mesial canals or at the nearest and most occlusal position for the distal canals. A multisegmented line was then continuously traced from the AF to the OR (Fig. 1).

The root canal length measured by tools in the software was noted as the CBCT length (CL). The CBCT estimations were performed twice at two-week intervals to test the intra-examiner reliability.

For the second part of the study, the CBCT data were opened in the dedicated software (3D Endo; Dentsply Sirona, Johnson, TN, USA) for analysis. The following steps were performed on the dedicated software. Three-dimensional images of the teeth were isolated from the surrounding material using software tools. The canal orifice and the AF of every canal were manually located. This step facilitated the creation of a semi-automatic line by the software. A series of adjustments was performed to ensure the most appropriate pathway of the canal in all three sagittal planes for the formation of a 3D reconstruction tooth image with the canal system inside and the K-files from the orifices to the apices. The proposed length was recorded as 3D-PL after the Suggest button was activated manually on the interface of the software. If a rubber stop position at the proposed length was not appropriate, its position could be manually amended for the best location; this length was recorded as the operator length (3D-OL). These estimations (3D-PL and 3D-OL) were performed three times and their averages were recorded. The dedicated software estimations were performed twice, with an interval of two weeks, to assess the intra-examiner reliability.

For the third part of the study, all teeth were removed from the plastic impression mould, rinsed with saline, dried with cotton and prepared for cavity access using access burs (Martin and Endo-Z; Dentsply Sirona, Ballaigues, Switzerland). After all canal orifices were observed, a #10 ISO K-file (K-File 010 21 MM; Dentsply Maillefer, Ballaigues, Switzerland) was inserted into all canals until the tip of the file was visible at the coronal margin of the AF using a stereomicroscope (Olympus SZX16; Olympus, Tokyo, Japan) at a magnification of 10×. The rubber stop on the shaft or the file was adjusted on the occlusal surface to obtain the reference point; the file was then removed from the canal and the length from the tip to apical surface of the rubber stop of the file was measured using a calliper (Mitutoyo; Mitutoyo, Kawasaki, Japan) and noted as the actual length (AL). The ALs were measured twice, with an interval of two weeks, to assess the intra-examiner reliability.

Teeth were then immersed in a freshly mixed alginate tray to measure root canal lengths using EAL (RomiApex A-15; Romidan, Kiryat Ono, Israel). One electrode of the EAL was embedded into the alginate material and the other electrode was attached to the metal shaft of a #10 ISO K-file. The #10 ISO K-file was then introduced into the canal until the apex mark lit up and stabilised for 5 s. The rubber stop was adjusted to the reference point on the occlusal surface, the K-file was removed from the canal and the length from the tip to apical surface of the rubber stop of the file was measured using a calliper. This length was noted as the electronic length (EL). The electronic measurements were the averages of three measurements. The electronic measurements were performed twice, with an interval of two weeks, to assess the intra-examiner reliability.

All data were stored and analysed using statistical software (MedCalc Statistical Software version 19; MedCalc Software, Ostend, Belgium). The data were first checked for normality with the Shapiro–Wilk test and the intra-examiner reliability was checked using the IntraClass Correlation (ICC) index. Fisher’s exact test, paired t-test and Bland–Altman plots were performed to analyse the data at a significance level of 0.05.

## Results

The proportions (%) of differences between the experimental endodontic length estimations with AL are displayed in Table 1. In the range of ± 0.5 mm, the EAL estimations gained the highest incidence of 100%. The lowest incidence was 38.4% from CBCT at a slice thickness of 2.4 mm.

**Table 1:** The incidence (%) of differences between the four methods and the AL measurements.

Groups	Shorter than AL N (%)		Equal to AL N (%)	Longer than AL N (%)		± 0.5 mm (%)
	> 0.5 mm	≤ 0.5 mm		≤ 0.5 mm	> 0.5 mm	
3D-PL–AL	8 (2.6)	46 (15.2)	23 (7.6)	214 (70.9)	11 (3.6)	93.8^a^
3D-OL–AL	4 (1.3)	164 (54.3)	27 (8.9)	105 (34.8)	2 (0.7)	98.0^b^
CL_0.6_–AL	48 (15.9)	47 (15.5)	0 (0.0)	73 (24.2)	134 (44.4)	${39.7}^{{\mathrm{c}}_{1}}$
CL_1.2_–AL	69 (22.8)	50 (16.6)	1 (0.3)	70 (23.2)	112 (37.1)	${40.1}^{{\mathrm{c}}_{2}}$
CL_2.4_–AL	42 (13.9)	34 (11.3)	1 (0.3)	81 (26.8)	144 (47.7)	${38.4}^{{\mathrm{c}}_{3}}$
EL–AL	0 (0.0)	218 (72.2)	54 (17.9)	30 (9.9)	0 (0.0)	100.0^d^

a,b,c_1,2,3_,dDifferent superscript letters indicate that there were significant differences at a level of 0.05 (${p}_{a,b}=0.013; {p}_{a,{c}_{\mathrm{1,2},3}}<0.001; {p}_{a,d}<0.001; {p}_{b,{c}_{\mathrm{1,2},3}}<0.001; {p}_{b,d}=0.030; {p}_{{c}_{1},{c}_{2}}=1.000;{p}_{{c}_{1},{c}_{3}}=0.802;{p}_{{c}_{2},{c}_{3}}=0.739; {p}_{{c}_{\mathrm{1,2},3},d}<0.001$*).*

3D-PL: Proposed length; 3D-OL: Operator length; EL: Electronic length; AL: Actual length.

CL_0.6_: CBCT length at slice thickness of 0.6 mm.

CL_1.2_: CBCT length at slice thickness of 1.2 mm.

CL_2.4_: CBCT length at slice thickness of 2.4 mm.

There were significant differences between the root canal length measured by the different experimental modalities and the AL (all *p* values were smaller than 0.05), but there were no significant (CL–AL) differences (*p* $=1.000, p=0.802 \mathrm{and} p=0.739$ for the differences between the (CL–AL) values at slice thicknesses of 0.6 and 1.2, 0.6 and 2.4 and 1.2 and 2.4 mm, respectively).

The mean bias, 95% CI, *p* values of the paired t-test and linear regression between AL and the experimental modalities are displayed in Table 2. The mean bias of the CBCT at a slice thickness of 1.2 mm was 0.131 mm and there was no fixed bias in this estimation ($p=0.129$, paired t-test in Bland–Altman analysis); therefore, the CBCT estimations at a slice thickness of 1.2 mm agreed with the AL. The remaining methods acquired fixed and/or proportional biases, so these remaining modalities disagreed with the AL.

**Table 2:** Mean biases, confidence intervals, *p* values in two statistical tests, fixed or proportional biases between experimental methods and AL measurements.

Groups	Paired t-test			Linear regression	Fixed bias	Proportional bias
	Mean bias	95% CI	*p*	*p*		
3D-PL–AL	-0.109	-0.138 to -0.080‏	< 0.001‏*	0.239	Yes	No
3D-OL–AL	0.026 ‏	0.008 to 0.045	0.006*	0.890	Yes	No
CL_0.6_–AL	0.346	0.218 to 0.473	< 0.001*	< 0.001*	Yes	Yes
CL_1.2_–AL	0.131	-0.038 to 0.301‏	0.129	< 0.001*	No	Yes
CL_2.4_–AL	0.468	0.343 to 0.593	< 0.001*	< 0.001*	Yes	Yes
EL–AL	0.050	0.042 to 0.058	< 0.001*	0.628	Yes	No

*Differences at significant level of 0.05.

3D-PL: Proposed length; 3D-OL: Operator length; EL: Electronic length; AL: Actual length.

CL_0.6_: CBCT length at slice thickness of 0.6 mm.

CL_1.2_: CBCT length at slice thickness of 1.2 mm.

CL_2.4_: CBCT length at slice thickness of 2.4 mm.

Further analysis of the data for the agreement between different root canal lengths measured by different modalities versus each other is displayed in Table 3. There was only agreement between the EL and CL at a slice thickness of 1.2 mm ($p=0.349,$ paired t-test in Bland–Altman analysis).

**Table 3:** Mean biases, confidence intervals, *p* values in two statistical tests, fixed or proportional biases among methods’ estimations.

Groups	Paired t-test			Linear regression	Fixed bias	Proportional bias
	Mean bias	95% CI	*p*	*p*		
3D-OL–3D-PL	-0.136	-0.158 to -0.113	< 0.001*	0.013	Yes	No
CL_0.6_–CL_1.2_	-0.214	-0.379 to -0.050	0.011*	0.483	Yes	No
CL_0.6_–CL_2.4_	0.122	0.039 to 0.205	0.004*	0.453	Yes	No
CL_1.2_–CL_2.4_	0.337	0.174 to 0.499	0.001*	0.764	Yes	No
EL–3D-OL	-0.024	-0.044 to -0.0030	0.025*	0.535	Yes	No
EL–3D-PL	-0.159	-0.190 to -0.129	< 0.001*	0.025	Yes	Yes
EL–CL_0.6_	0.296	0.167 to 0.424	< 0.001*	0.342	Yes	No
EL–CL_1.2_	0.081	-0.089 to 0.251	0.349	0.951	No	No
EL–CL_2.4_	0.418	0.292 to 0.543	< 0.001*	0.647	Yes	No

*Differences at significant level of 0.05.

3D-PL: Proposed length; 3D-OL: Operator length; EL: Electronic length; AL: Actual length.

CL_0.6_: CBCT length at slice thickness of 0.6 mm.

CL_1.2_: CBCT length at slice thickness of 1.2 mm.

CL_2.4_: CBCT length at slice thickness of 2.4 mm.

## Discussion

The results of the present study reveal that there was an only agreement between CL at a slice thickness of 1.2 mm and the AL; therefore, the null hypothesis was partially rejected.

The results show that, in the four modalities evaluated, only CBCT agreed with the AL measurements with the lowest mean bias and without proportional bias. The best accuracy of the measurements in the range of ± 0.5 mm was with the EAL, although this device disagreed with the AL.

The continuing development of new modern endodontic instruments^22,23^, such as rotary files or electronic apex locators, plays an important role in efforts to achieve better endodontic outcomes, even with resorbed primary teeth^24^. The dedicated software was developed for endodontic therapy in the clinical setting. WL determination is one of the most creative features of the dedicated software, with the option of manually changing WL by the operator whenever they want. This feature was developed in an effort to maximally reduce operator errors in WL determination. Depending on the curvature of the canal, the operator can estimate the length of the prepared canal to adjust the WL in the final steps. The virtual pathway of the canal is intuitively displayed on the screen, which assists in effective visualisation and management of the root canal preparation. One of the most important requirements for this special software is that the voxel size of the CBCT images should be below 0.2 mm. The present study used the lowest voxel size of the CBCT device in order to obtain maximum accuracy of the software. However, the results show that although the mean bias of the differences between the dedicated software measurements and the AL was reduced significantly after correcting the working length, the difference between 3D-OL and AL measurements was still considerable, meaning that the 3D-OL disagreed with the AL measurements.

Extracted human teeth are commonly used for studies using CBCT in WL determinations in dry mandibles or jaw models^25–27^. The insertion of many teeth in an impression tray may induce certain artifacts because of the crowding of teeth in a small space. However, anatomical landmarks are defined easily and exactly with no interferences from clear CBCT images. Although extracted human teeth seem to be appropriate for assessment of the precision of endodontic length determinations using CBCT images, artificial training teeth can completely meet the demands of this goal^28^. The actual root canal length of the artificial tooth was selected as the gold standard in evaluating the accuracy of the CBCT WL measurements^28^. Accurate 3D endodontic length measurements can be achieved with the dedicated software. However, the WL has to be checked, controlled and maintained continuously during the instrumentation phase to define changes, especially in extremely curved canals^28^.

The results of the present study show that, in the range of ± 0.5 mm from the apical foramen, the EAL measurements were the best tool for root canal length determination. This result agrees with those of previous studies^3,18–20^ and, even in the more difficult situation, the preciseness of the EAL was still high^24^.

The freshly mixed alginate tray for the immersion of teeth to facilitate electronic length measurements in the present study was a simple and successful model that had been used in previous studies^18–20^. More complicated models should be created to help to further research in root canal length determination and preparation. Additional investigations should be performed using an integrated EAL in endodontic motor and simultaneous root canal instrumentation to approach the clinical situation more closely.

CBCT is an additional method for the determination of WL and is valuable for retreatment when removing gutta-percha to save time and prevent over-instrumentation^8^. Significant artifacts from heavily metallic restorations are the major drawbacks when using 3D images for WL determination^8^. More artifacts mean a greater approximate range of length, with data providing only an estimate of the actual length^8^.

CBCT scans depend on many parameters, such as voxel size, field of view, electric current intensity, reconstruction algorithm, beam collimation and slide thickness^11^. Slice thickness affects the resolution of the image, leading to an impact on the accuracy of measurements^11^. The slice thickness is inversely proportional to image noise and decreasing the slice thickness increases the latter^14^. A common sense approach is that a thin, magnified slice supplies increasingly better documentation; however, this concept is not sufficiently supported by current knowledge^14^. Most often, a thicker slice increases the contrast of the image and might provide better identification of the volume of interest^12^. However, previous studies have investigated the optimal slice thickness for different purposes and the results have not been consistent^11–16^. These studies used the same platform as in the present study^18–20^; however, only one mentioned a slice thickness of 1 mm for CBCT measurements^18^. The results of the present study reveal that a slice thickness of 1.2 mm was optimal under the conditions of this study.

Following the ALARA principle (as low as reasonably achievable), the patient should be exposed to the lowest amount of radiation needed for the examination of any condition^7^. Therefore, CBCT scans should be carefully indicated; other modalities have been considered as a replacement. The same principle has been suggested by the American Association of Endodontists^29^. Therefore, CBCT could not be used as a routine diagnostic procedure in endodontics and patient exposure to CBCT only for the determination of root canal length is not recommended^28^. For patients with pre-existing CBCT, dedicated software is very useful for endodontic planning and other necessary procedures, such as the evaluation of anatomical structures and the measurement of WL^28^.

The present study was performed at only one voxel size of 0.075 mm with three different slice thicknesses; the platform did not support periapical digital radiographs. Further investigations should be performed to evaluate more voxel sizes, different slice thicknesses and more suitable platforms for periapical radiographs.

## Conclusions

The CBCT at the smallest slice thickness estimation was not the best modality, which is in agreement with the AL. The EAL was an accurate and reliable method for root canal length measurement.
